# General Practitioners and Their Fees

**Published:** 1903-05-02

**Authors:** 


### General Practitioners and Their Fees.

It is an old tradition in the medical profession, begotten of the time when the custom was for the doctor to render a bill of inordinate length, that it usually found its way to the bottom of the patient's file. The average patient treated the doctor's bill as a claim which could be met at any time, or be put off indefinitely. We fear this type of patient is still to be met with. Up and down the country various idiosyncrasies remain in regard to these matters. In some large towns, especially in the Midlands, patients expect a discount off their doctor's bill. Some of them insist upon every item being separately set forth ; whilst others again grumble where the items are given, and prefer a mere statement: For Professional Services £——. A wide experience of medical practice in all its aspects has brought us to the conclusion that the business side of the matter needs closer attention nowadays than formerly. After all, medical practice entails the necessity to provide substantial sums every week in cash for the payment of working expenses. It follows, that every qualified medical man who aspires to do a large and increasing practice, must either be possessed of capital, or so arrange his business that in addition to book debts, he shall have a number of patients who pay his fees in cash as and when the work is done.

The difficulties which beset medical practitioners in this country are in no wise exceptional. The *Daily Mail* of the 23rd instant published an article from a correspondent in Berlin which pointed out that the German doctors under the Invalids Insurance Law, which is controlled by the Government, only receive about 2d. per visit per patient from the managers of the sick funds. These payments are provided by compulsory contributions from employers and employed alike. A movement is being organised throughout Germany, which, if persisted in, will cause all the physicians there who are employed by sick fund societies to strike on the 1st July next. The object of the strike appears to be the appointment of an impartial committee to fix a minimum fee according to the conditions existing in the respective localities, and to empower the patients to select their own doctors, instead of leaving the choice of the physician, as at present, to the managers of the sick funds. The Government has introduced a measure before the Reichstag which appears to have excited the wrath of the medical profession, who regard it as unsatisfactory. In England contract practice has always been more or less a bone of contention. We recently exposed the scandalous under-payment of the doctors who attend the crofters under contract with the local authorities. In London and some other large towns there is a class of practitioners, who have established cheap dispensaries in connection with their own surgeries, which are regarded as a grave scandal by some members of the profession. We have also to contend with the so-called medical associations, *i.e.*, organisations of the working-classes, which endeavour to secure the medical aid they need by contracting with one or two members of the profession to give their whole time for a fixed salary. It must be admitted that although in a few instances contract practice works satisfactorily to all parties concerned, speaking generally, it is correct to say that this branch of professional work rests to-day in England upon no more satisfactory basis than it does in Germany and elsewhere.

In a large town it is seldom difficult for a practitioner of intelligence to gradually build up a large ready-money practice, profitable to himself and popular with his patients. Many club doctors take their clubs at such a low rate that the work must be done at a loss. Some sixty years ago this fact was fully recognised in some of the large Midland towns, and so-called self-supporting dispensaries were established, in connection with the surgeries of the leading general practitioners, which proved beneficial to all parties. It was found that to book an attendance and medicine to patients below a certain social grade was simply to cumber the books with a number of accounts, the majority of which were seldom or never paid. The self-supporting dispensaries met this defect and applied an effectual remedy. Each patient on first attendance at the surgery paid 3s. 6d. for one week's attendance and medicine. If the call came after 10 a.m. in the morning and the case had to be visited that day, the patient paid 3s. 6d. extra for a special visit. There were practically no other regulations, the system was widely popular, and brought a considerable sum each week in ready money to the practice. We are confident, it pays no member of the profession to accept less than 4s. a head per annum for contract work, and we consider 6s. a head the minimum which ought to be taken. We further feel confident, that instead of the sick clubs, the general adoption of the self-supporting dispensary idea as we have explained it, would be advantageous to patients and practitioners alike. Whenever the medical practitioner can arrange the fee directly with his patient, the more certainty will there be that satisfaction will result to all parties. Hence the plan referred to is infinitely preferable to contract work and offers many substantial advantages.

